# Cdc6 ATPase activity disengages Cdc6 from the pre-replicative complex to promote DNA replication

**DOI:** 10.7554/eLife.05795

**Published:** 2015-08-25

**Authors:** FuJung Chang, Alberto Riera, Cecile Evrin, Jingchuan Sun, Huilin Li, Christian Speck, Michael Weinreich

**Affiliations:** 1 Van Andel Research Institute, Grand Rapids, United States; 2 Faculty of Medicine, Hammersmith Hospital Campus, Imperial College London, London, United Kingdom; 3 Biosciences Department, Brookhaven National Laboratory, New York, United States; 4 Department of Biochemistry and Cell Biology, Stony Brook University, New York, United States; Harvard Medical School, United States

**Keywords:** DNA replication, ORC/Cdc6, helicase loader, AAA+ ATPases, *S. cerevisiae*

## Abstract

To initiate DNA replication, cells first load an MCM helicase double hexamer at origins in a reaction requiring ORC, Cdc6, and Cdt1, also called pre-replicative complex (pre-RC) assembly. The essential mechanistic role of Cdc6 ATP hydrolysis in this reaction is still incompletely understood. Here, we show that although Cdc6 ATP hydrolysis is essential to initiate DNA replication, it is not essential for MCM loading. Using purified proteins, an ATPase-defective Cdc6 mutant ‘Cdc6-E224Q’ promoted MCM loading on DNA. Cdc6-E224Q also promoted MCM binding at origins in vivo but cells remained blocked in G1-phase. If after loading MCM, Cdc6-E224Q was degraded, cells entered an apparently normal S-phase and replicated DNA, a phenotype seen with two additional Cdc6 ATPase-defective mutants. Cdc6 ATP hydrolysis is therefore required for Cdc6 disengagement from the pre-RC after helicase loading to advance subsequent steps in helicase activation in vivo.

**DOI:** http://dx.doi.org/10.7554/eLife.05795.001

## eLife digest

Before a cell divides, it first creates copies of its DNA so that the two daughter cells both receive a complete copy of its genetic blueprint. The DNA is arranged in a double helix that is made of two single DNA strands that twist together. The process of copying the DNA requires a group or ‘complex’ of proteins called the MCM helicase complex that binds to this double-stranded DNA molecule. MCM then separates the two DNA strands to allow the production of new DNA strands in a process that uses the original strands as templates. After copying, the two resulting DNA double helices each have one of the original strands and one new strand.

An enzyme called Cdc6 works together with several other proteins to help MCM bind to double-stranded DNA. Cdc6 uses energy to promote DNA copying, but it is not clear how this works. Here, Chang et al. studied the activity of yeast Cdc6. A mutant form of Cdc6 that lacked its enzyme activity still promoted MCM binding to DNA. However, yeast cells with this mutant enzyme were unable to copy their DNA and did not divide.

Next, Chang et al. used a technique called ‘single particle electron microscopy’ to investigate how the MCM complex, DNA and Cdc6 interact with each other. These experiments show that normal Cdc6 enzymes detach from the MCM complex after the energy is used to allow DNA copying and cell division to proceed. However, the mutant Cdc6 enzymes remain stuck to the complex, which blocks DNA copying. In cells, if the mutant Cdc6 enzymes are deliberately destroyed after the MCM complex binds to DNA, DNA copying proceeds normally. This implies that Cdc6 inhibits MCM activity as long it remains bound to the complex.

A similar sequence of steps occurs when helicases bind to DNA in bacteria, which suggests that this important process has been maintained during billions of years of evolution. The next steps will be to understand how Cdc6 is able to inhibit the MCM complex, and how Cdc6's enzyme activity enables it to detach from the complex later on.

**DOI:** http://dx.doi.org/10.7554/eLife.05795.002

## Introduction

Eukaryotic cells allow pre-replicative complex (pre-RC) assembly at origins only once per cell cycle (Li and Araki, 2013; Yardimci and Walter, 2014) to promote genome integrity (Abbas et al., 2013). An inactive MCM (mini-chromosome maintenance) double hexamer is first loaded on double-stranded DNA (dsDNA) in late M- to early G1-phase and then activated in a subsequent step that requires conserved protein kinases (Labib, 2010). Cdc6 is a AAA+ ATPase family member that binds ORC (origin recognition complex), and together these proteins function as an MCM ‘helicase loader’ (Liang et al., 1995; Cocker et al., 1996; Neuwald et al., 1999; Wendler et al., 2012; Duderstadt and Berger, 2013). Since the weakly hydrolyzed ATP-γ-S analogue traps an OCCM complex on DNA containing ORC-Cdc6-Cdt1 and a single hexamer of MCM (all six MCM subunits are also AAA+ proteins), double hexamer loading requires ATP hydrolysis (Sun et al., 2013). Following MCM complex loading, Cdt1 and Cdc6 disengage from the pre-RC to allow additional protein loading complexes to activate the MCM helicase (Yardimci and Walter, 2014).

**Figure 1.**
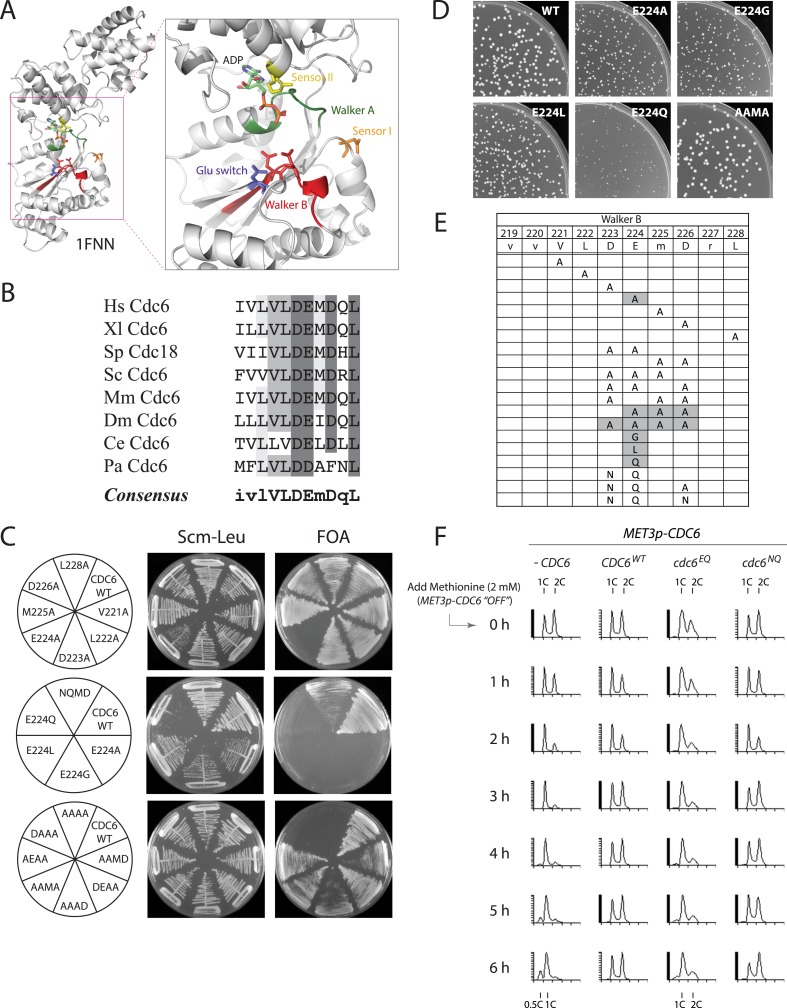
The Cdc6 Walker B catalytic residue is essential in yeast but is easily bypassed by intragenic suppressor mutations. (**A**) Structure of the *Pyrobaculum aerophilum* Cdc6 orthologue with bound ADP (Liu et al., 2000) highlighting the ATP binding pocket and key residues. The ‘DD’ Walker B residues are colored red and the sensor I residue is colored orange. (**B**) Multiple sequence alignment of Cdc6 Walker B motif. Uppercase letters in consensus are highly conserved. From top to bottom: *Homo sapiens*, *Xenopus laevis*, *S. pombe*, *S. cerevisiae*, *Mus musculus*, *Drosophila melanogaster*, *C. elegans*, and *Pyrobaculum aerophilum* Cdc6 homologues. (**C**) Viability analysis of Cdc6 Walker B motif mutants reveals only E224 is essential. M4466 (*cdc6Δ::ura3* pRS416-*CDC6*) was transformed with the indicated pMW71-derived plasmids (listed in Supplementary file 2) and then restreaked onto SCM-Leu plates representing growth of *CDC6/cdc6* or FOA plates, growth of *cdc6* mutant only. (**D**) Growth of plasmid *cdc6* derivatives transformed into wild-type yeast (W303-1A) reveals that multiple E224 substitutions have dominant growth affects. (**E**) Summary of Cdc6 mutational analysis. Shaded regions are non-viable mutants. (**F**) Flow cytometry profiles of strains M4759 (K4055 × pRS415, vector), M4758 (K4055 × pMW71, *CDC6-WT*), M4760 (K4055 × pFJ21, *cdc6-EQ*), and M4762 (K4055 × pFJ230, *cdc6-NQ*) after addition of methionine to repress expression of wild type *MET3p-CDC6* present in all strains. **DOI:** http://dx.doi.org/10.7554/eLife.05795.003

The AAA+ family shares multiple conserved motifs, including Walker A and B motifs that promote ATP binding and hydrolysis, as well as sensor 1 and 2 motifs that help couple changes in ATP/ADP occupancy with protein conformational changes (Wendler et al., 2012; Duderstadt and Berger, 2013) (Figure 1A). Early mutational analysis of the Walker A motif agreed that budding yeast Cdc6 must bind ATP to execute its essential function (Perkins and Diffley, 1998; Wang et al., 1999; Weinreich et al., 1999). However, there have been conflicting reports on the role of Cdc6 ATPase activity in pre-RC assembly and yeast viability. Two different mutants altering the Walker B motif (required for ATP hydrolysis) gave opposite phenotypes: a Cdc6-E224G mutant was lethal and acted as a dominant negative mutant when overexpressed (Perkins and Diffley, 1998), but a double Cdc6-D223A, E224A mutant was viable suggesting the Cdc6 ATP hydrolysis might not be required for its essential function (Weinreich et al., 1999). A Walker B DExx>AAxx mutant in *Schizosaccharomyces pombe* Cdc6 (Cdc18) was also shown to be viable (Liu et al., 2000). A polar residue in the sensor 1 motif at the tip of the β4-strand (corresponding to N263 in budding yeast Cdc6) is also required for ATP hydrolysis in various AAA+ proteins (Wendler et al., 2012). Mutation of Cdc6 N263 to A has been reported to result in normal growth at 24°C and no growth at 37°C in one study (Schepers and Diffley, 2001) but was lethal at 30°C in another (Takahashi et al., 2002). Alanine mutation of the Cdc6 sensor 2 arginine (R332) had a modest effect on growth, suggesting it does not perform an essential role (Schepers and Diffley, 2001), but mutation of R332 to E was lethal (Takahashi et al., 2002). Lastly, AAA+ members typically form multisubunit protein assemblies and an ‘R-finger’ residue in one subunit can stimulate ATP hydrolysis in an adjacent subunit. The R-finger is named for an analogous arginine first discovered in the Ras GTPase-activating protein, p120^GAP^ (Scheffzek et al., 1997). However, a triple alanine mutant spanning the budding yeast Cdc6 R-finger (R274) gave no growth phenotype (Schepers and Diffley, 2001), suggesting that the Cdc6 R-finger is not required for stimulating either ORC or MCM ATPase activity.

**Figure 1—figure supplement 1.**
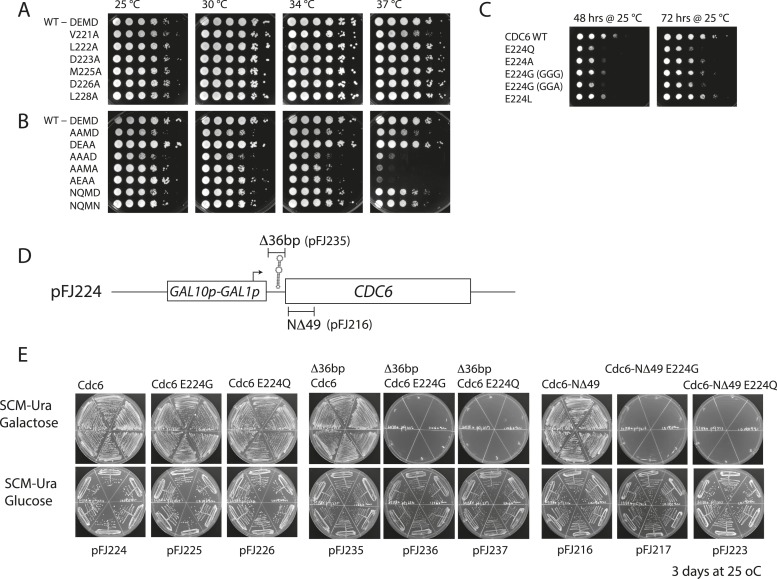
Growth properties of various *cdc6* Walker B mutants. For (**A**) and (**B**), M4466 (W303-1A, *cdc6*Δ::*ura3*) containing only the indicated *cdc6* alleles on pMW71 (Supplementary file 2) were spotted onto YPD plates in a 10-fold dilution series and incubated at the indicated temperatures. (**A**) Alanine scanning mutants across the Cdc6 Walker B box. (**B**) Double and triple mutants within the ‘DEMD’ core region. The AAAD, AAMA, and AEAA mutants exhibited substantial temperature sensitivity. (**C**) The indicated Cdc6-E224 mutant alleles or wild-type *CDC6* on pMW71 (Supplementary file 2) were transformed into wild-type yeast (W303-1A). These transformants were spotted using 10-fold serial dilutions onto SCM-Leu plates (selecting for the pMW71-derivative) to quantitatively measure their dominant negative growth phenotype. This indicates that all the E224 alleles exhibited some dominate growth effects over the wild type. Two separate codons for E224G were tested. (**D**) Diagram of pGAL-CDC6 plasmids. pFJ224 contains the *GAL1,10* promoter 39 bp upstream of wild-type *CDC6* coding sequence to give a *GAL1p-CDC6* promoter fusion. This plasmid contains a 49-bp sequence (CCGGGAATTTCCGGTGGTGGTGGTGGAATTCTAGACTCC ATG TCA GCT A) predicted to form a stable multi-stem loop structure that overlaps the Cdc6 ATG, underlined. The 39 bp immediately preceding the Cdc6 ATG is derived from vector sequences. This pGAL-Cdc6 construct complements Cdc6 function when yeast is propagated on galactose but not on glucose media. (**E**) Overexpression of Cdc6-E224G or -E224Q mutant proteins from pFJ216 (differing from pFJ224 only by a deletion of Cdc6 residues 2–49, which significantly stabilizes Cdc6 protein) or from pFJ235 (differing from pJF224 only by a 36 bp deletion disrupting the stem loop) on galactose causes complete growth inhibition of wild type yeast. In contrast, galactose-induced expression of these mutant proteins in the pFJ224 plasmid background causes only a mild growth inhibition, indicative of lower Cdc6 protein induction. The indicated plasmids (Supplementary file 2) were transformed into wild-type yeast (W303-1A), and 6 transformants each were streaked onto selective minimal media (SCM-Ura) containing galactose or glucose as the carbon source and incubated at 25°C for 3 days. **DOI:** http://dx.doi.org/10.7554/eLife.05795.004

An early in vitro MCM loading assay utilizing purified proteins together with crude extracts reported that Cdc6 ATP hydrolysis (using the Cdc6-E224G mutant) was required for MCM loading (Randell et al., 2006). However, two recent reports using only purified proteins (and using Cdc6-E224G or Cdc6-N263A mutants) find that Cdc6 ATP hydrolysis is not required for efficient MCM loading in vitro (Coster et al., 2014; Kang et al., 2014). The Cdc6-N263A sensor 1 mutant was previously shown to be defective in ATP hydrolysis in vitro (Speck and Stillman, 2007). Coster et al. and Kang et al. have suggested that Cdc6 ATPase is required for a quality control step that ejects incomplete or non-functional MCM hexamers before loading. Surprisingly, no systematic mutational analysis of the Cdc6 Walker B residues has been reported.

Here, we have combined yeast genetics and in vivo assays together with an in vitro MCM loading reaction to probe the role of Cdc6 Walker B motif residues for viability and growth, ATP hydrolysis, pre-RC assembly, and DNA replication in yeast. We find that Cdc6 ATP hydrolysis is required for yeast viability but not for MCM loading either in vitro or in vivo. Furthermore, since the requirement for Cdc6 ATP hydrolysis can be bypassed by degrading Cdc6 ATPase defective proteins after MCM loading, ATP hydrolysis is likely instead required to disengage Cdc6 from the pre-RC (presumably an ORC-Cdc6-MCM intermediate) to allow subsequent steps in MCM helicase activation.

## Results and discussion

The Walker B motif (DExx) contains a glutamate residue that is required for the hydrolysis of ATP to ADP (Ogura et al., 2004) and is highly conserved among Cdc6 orthologues (Williams et al., 1997) (Figure 1A,B). An alanine scan across the yeast Cdc6 Walker B motif revealed that only the E224A mutation caused inviability (Figure 1C, top panel). Mutation of E224 to G (Perkins and Diffley, 1998), L, or Q also caused inviability (Figure 1C, middle panel). The other single alanine mutants with the Walker B motif exhibited nearly wild type growth over a wide temperature range (Figure 1—figure supplement 1). Expression of plasmid-borne *cdc6-E224G*, *-A*, *-L*, and *-Q* mutants from the Cdc6 promoter showed that all mutants were dominant negative for growth in otherwise wild-type yeast cells, with the E224Q mutant having the greatest effect (Figure 1D; Figure 1—figure supplement 1). Interestingly, all double and triple mutant combinations that changed the preceding aspartate D223 together with E224A or E224Q restored viability, including a triple mutant that removed all acidic residues from the Cdc6 DEMD Walker B motif (Figure 1C, bottom panel). The growth properties of the double and triple mutants were surprisingly robust with the exception of the triple alanine mutants, ‘AAAD’, ‘AAMA’, and ‘AEAA’, which were temperature sensitive at 37°C (Figure 1—figure supplement 1). The results of the mutational scan are summarized in Figure 1E. These data show that Cdc6 E224 is the only residue in the Walker B motif required for viability. Furthermore, the relative ease with which the requirement for the E224 catalytic residue could be bypassed by the intragenic suppressor mutations suggested that ATP hydrolysis might not be mechanistically coupled to MCM loading.

To determine whether the Cdc6-E224Q mutant exhibited a cell cycle block, we propagated cells using a heterologous *MET3p-CDC6* promoter fusion (Piatti et al., 1995) and then measured DNA content using *CDC6* plasmid alleles expressed from the *CDC6* promoter after shutting off *MET3p-CDC6* expression with methionine addition. The *CDC6* and *cdc6-DE(223,224)NQ* (or ‘*NQ*’) strains proliferated normally after repressing wild-type *MET3p-CDC6* expression (Figure 1F). However, the *cdc6-E224Q* mutant strain had a larger fraction of G1- and S-phase cells even at t = 0 hr (when both wild type and mutant Cdc6 were expressed) and largely arrested in G1-phase by t = 6 hr after shutting off wild-type Cdc6 expression, as did the cells lacking any additional copy of *CDC6*. This indicates that *cdc6-E224Q* blocks progression into S-phase as expected for a defect in DNA replication initiation.

**Figure 2.**
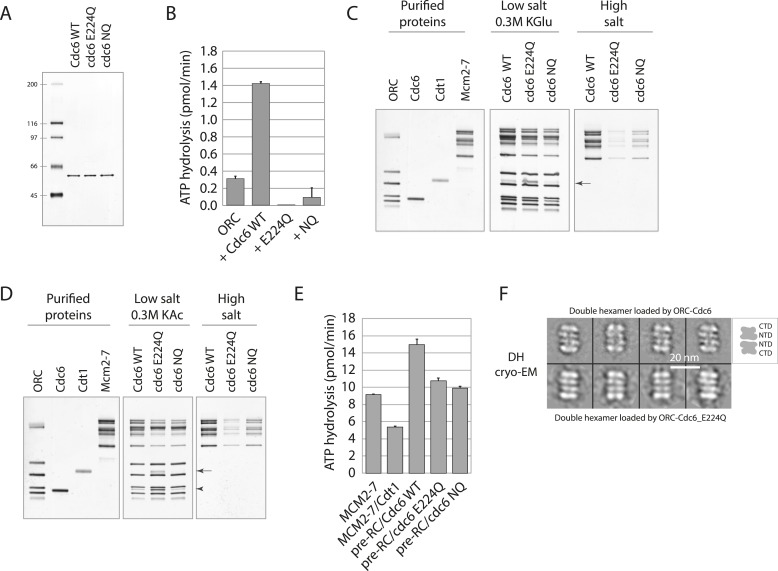
Cdc6 ATPase activity is not required for MCM loading in vitro. (**A**) Silver-stained 10% SDS gel with molecular weight standards and 100 ng of the indicated purified Cdc6 proteins. (**B**) Cdc6 ATPase assays. (**C**) MCM loading assay using the purified proteins shown on left. ‘Low salt’ shows proteins associated with DNA, ‘High salt’ wash reveals loaded MCM protein. Arrow marks Cdt1. (**D**) MCM loading assay as in (**C**) using a more stringent (0.3 M K acetate) low salt wash indicates that Cdc6-E224Q is stabilized on DNA relative to WT Cdc6 and Cdc6-NQ protein. Arrow marks Cdt1, arrowhead marks Cdc6. (**E**) ATPase assays with Cdc6-WT-ORC-DNA, Cdc6-E224Q-ORC-DNA, or Cdc6-NQ-ORC-DNA complexes after MCM-Cdt1 addition. The first two lanes show soluble MCM and MCM-Cdt1 ATPase activities as controls. (**F**) The double hexamer of Mcm2-7 loaded by Cdc6-E224Q after high salt wash (1 M NaCl) of loading reactions is indistinguishable from that loaded by wild-type Cdc6. 528 raw cryo-EM particle images were used for 2D classification and averaging for the dhMCM protein loaded with ORC-Cdc6-E224Q. 3217 raw particles were used to generate the averaged views of the dhMCM loaded with wild-type ORC-Cdc6. **DOI:** http://dx.doi.org/10.7554/eLife.05795.005

We purified Cdc6-WT, Cdc6-E224Q, and Cdc6-NQ proteins from bacteria (Figure 2A) and then measured their ATPase activity. Cdc6 has no ATPase activity when assayed alone but its ATPase activity is measurable in the context of an ORC-Cdc6-DNA complex as an *increase* in ATP hydrolysis seen by ORC-DNA alone (Randell et al., 2006). ORC had a low intrinsic ATPase activity on DNA but the addition of wild-type Cdc6 increased the overall ATPase activity by 4–5 fold, as seen previously (Randell et al., 2006) (Figure 2B). In contrast, the Cdc6-E224Q or -NQ mutant proteins had no ATPase activity (Figure 2B) because they did not increase the ORC–DNA ATPase activity but instead suppressed the minimal ORC ATPase activity. This analysis also reveals that the NQ mutant promotes yeast viability without restoring Cdc6 ATPase activity.

**Figure 2—figure supplement 1.**
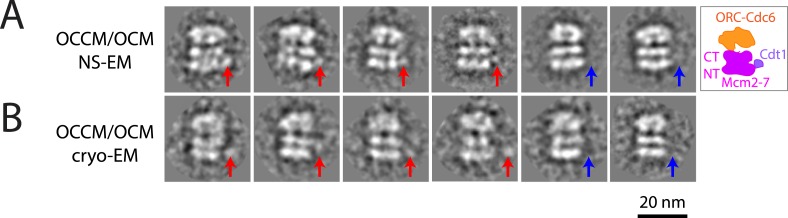
MCM loading by ORC-Cdc6-E224Q gives rise to a heterogeneous mixture of intermediates by EM. (**A**) Negative stain-EM and (**B**) cryo-EM of complexes formed with ORC, Cdc6-E224Q, and MCM-Cdt1 proteins revealed a heterogeneous mixture of OCCM (ORC-Cdc6-Cdt1-MCM) and OCM (ORC-Cdc6-MCM) complexes; averages from 3655 individual particle images and 1436 cryo-EM raw particle images, respectively. The red arrows point to Cdt1 density, and blue arrows indicate the absence of Cdt1. The structure of Cdc6-E224Q-containing OCCM particles is similar to wild-type OCCM assembled in the presence of ATP-γ-S. However, the Cdt1 density is variable in its location in the mutant OCCM and is of course, absent in OCM complexes. Most of the particles were OCCM. We do not know the exact ratio between OCCM and OCM because the views without Cdt1 density are not necessarily of OCM; they could be OCCM particles at slightly different side views that prevented visibility of Cdt1. **DOI:** http://dx.doi.org/10.7554/eLife.05795.006

We next examined the ability of wild type and mutant Cdc6 proteins to load MCM using an in vitro assay with purified proteins (Evrin et al., 2009). ORC, Cdc6 (wild type or mutant), Cdt1, and MCM were incubated at 24°C with replication origin-containing plasmid DNA linked to magnetic beads and then washed under low salt conditions. This allows detection of proteins specifically bound to origin DNA. Wild type and both mutant Cdc6 proteins bound to ORC and promoted binding of MCM to DNA (Figure 2C, low salt). Interestingly, the E224Q mutant had a higher amount of Cdt1 bound than the wild type or Cdc6-NQ complex, as reported for a Cdc6-E224G mutant (Randell et al., 2006; Fernandez-Cid et al., 2013), suggesting build up of an intermediate Cdt1-containing complex prior to dhMCM (double hexamer MCM) loading. ‘Loaded’ dhMCM that encircles dsDNA is resistant to a 0.5 M high salt wash but ORC, Cdc6, Cdt1 and any MCM proteins that are merely ‘associated’ with DNA are not. Both mutant Cdc6 proteins were capable of loading MCM protein, albeit less efficiently than wild-type Cdc6 (Figure 2C, high salt). The increased Cdt1 retention in low salt by Cdc6-E224Q was also seen in reactions treated with a more stringent (0.3 M K acetate) low salt wash (Figure 2D). In addition, Cdc6-E224Q protein was retained within the low salt complex but wild-type Cdc6 and the Cdc6-NQ protein were significantly less stable (Figure 2D, Cdc6 is marked with an arrowhead). Cdc6-E224G is also defective in dissociating from the pre-RC relative to wild-type Cdc6 (Coster et al., 2014). Cdc6 ATP binding is required for interaction with ORC and for pre-RC assembly (Perkins and Diffley, 1998; Weinreich et al., 1999; Speck et al., 2005) and since Cdc6-E224Q and Cdc6-NQ proteins formed similar pre-RC complexes to wild-type Cdc6 (Figure 2C,D, low salt), they are not defective in ATP binding.

Although ORC-Cdc6-E224Q complexes exhibited no ATPase activity (Figure 2B), substantial ATPase activity was evident after MCM and Cdt1 protein addition (Figure 2E), at about two-thirds the level of the wild-type reaction (Fernandez-Cid et al., 2013). Therefore, Cdc6-E224Q does not prevent MCM ATPase activity, which might be sufficient to support double hexamer loading. Recent experiments using multiple ATP binding and ATPase-defective MCM mutants in yeast support the model that MCM ATPase activity is necessary for efficient double hexamer loading (Coster et al., 2014; Kang et al., 2014).

Using cryo-EM, the Mcm2-7 double hexamer loaded by ORC and mutant Cdc6 has the characteristic four-layered architecture in side views comprised of the two distal C-terminal AAA layers and the two middle N-terminal layers from two Mcm2-7 hexamers in their side views. This structural organization is identical to the double hexamer loaded by the wild-type ORC-Cdc6 (Figure 2F). As mentioned previously, ATP-γ-S traps a normally transient ORC-Cdc6-Cdt1-MCM (OCCM) intermediate on the DNA with a single MCM hexamer encircling dsDNA but with a small gap in the hexamer at the Mcm2-Mcm5 interface (Bochman and Schwacha, 2010; Costa et al., 2011; Sun et al., 2013; Samel et al., 2014). OCCM complexes are not seen with wild-type proteins in ATP, since ATP hydrolysis quickly promotes Cdt1 release to give a ‘OCM’ intermediate complex and then double hexamer loading (Yardimci and Walter, 2014). Both negative staining-EM and cryo-EM revealed a heterogeneous mixture of OCCM and OCM complexes formed with the Cdc6-E224Q protein in the presence of ATP (Figure 2—figure supplement 1) that were not seen with wild-type Cdc6. These data also suggest that Cdc6 ATP hydrolysis promotes Cdc6 and Cdt1 release from the pre-RC (Speck et al., 2005; Fernandez-Cid et al., 2013) since these OCCM and OCM reaction intermediates accumulate with the Cdc6-E224Q mutant. Taken together, the biochemical data show that the Cdc6-E224Q protein lacks ATPase activity, is defective in Cdc6 release from the pre-RC, but can promote dhMCM loading onto DNA.

**Figure 3.**
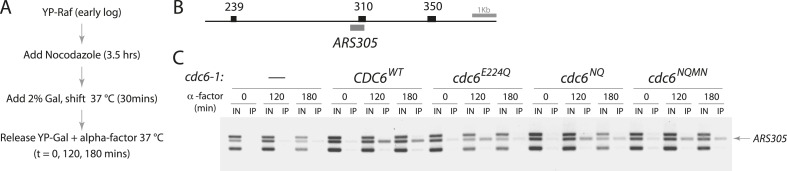
Cdc6 ATPase mutants promote MCM origin binding in yeast. (**A**) Cell synchronization protocol for MCM ChIP. (**B**) PCR products amplified in base pairs surrounding *ARS305.* (**C**) All the *cdc6* ATPase mutants tested promote MCM binding to origins similar to the wild-type *CDC6* regardless of whether they complement yeast viability. Strains used: M378 (*cdc6-1*), M4455 (*cdc6-1 GAL1p-CDC6::LEU2*), M4531 (*cdc6-1 GAL1p-cdc6-E224Q::LEU2*), M4513 (*cdc6-1 GAL1p-cdc6-NQ::LEU2*) and M4464 (*cdc6-1 GAL1p-cdc6-NQMN::LEU2*). **DOI:** http://dx.doi.org/10.7554/eLife.05795.007

We used MCM ChIP to test whether Cdc6-E224Q could promote MCM binding to ARS sequences in vivo. Since Cdc6-E224Q expression was dominant to the wild type, we arrested cells expressing only *cdc6-1* (a temperature-sensitive *cdc6* allele) in G2/M using nocodazole at 25°C, shifted cells to 37°C, and simultaneously turned on expression of an additional wild-type or mutant Cdc6 protein under the control of the Gal1 promoter. Cells were then released from the G2/M phase block at 37°C in the presence of galactose and harvested during a G1-phase pheromone block for MCM ChIP (Figure 3A,B). Although MCM protein was not detected at *ARS305* in the nocodazole arrest or in the absence of Cdc6 expression, all three mutant proteins (E224Q, NQ, and NQMN) promoted MCM binding to *ARS305*, as did wild-type Cdc6 (Figure 3C).

**Figure 4.**
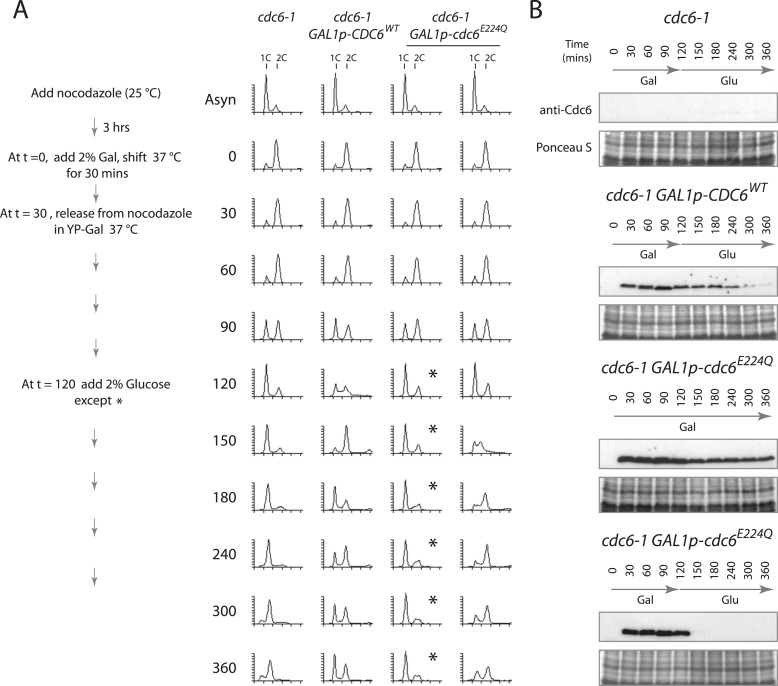
Cdc6 removal after MCM loading bypasses ATPase requirement and is sufficient to allow DNA replication. (**A**) Synchronization protocol (left) and flow cytometry profiles (right) of yeast strains M378 (*cdc6-1*), M4455 (*cdc6-1 GAL1p-CDC6::LEU2*), and M4531 (*cdc6-1 GAL1p-cdc6-E224Q::LEU2*). Asynchronous cells were arrested in G2/M for 3 hr with nocodazole, shifted up to 37°C for 30 min (from t = 0 to 30 min), and then released into G1-phase at 37°C expressing no additional Cdc6, *GAL1p-CDC6* or *GAL1p-cdc6-E224Q*. *GAL1* promoter-driven *CDC6* expression was shut off at t = 120 min by the addition of glucose except where marked by an asterisk. Identical flow profiles to M4531 were seen using an independent *cdc6-1 GAL1p-cdc6-E224Q* integrant strain, M4530. (**B**) Cdc6 Western blots (top panels) and total protein (bottom panels) by Ponceau S staining of the samples are shown in panel **A**. The addition of glucose at 120 min causes Cdc6 wild-type protein to disappear and this occurs more rapidly for the Cdc6-E224Q mutant. **DOI:** http://dx.doi.org/10.7554/eLife.05795.008

To test whether the MCM protein loaded by Cdc6-E224Q could promote DNA replication, that is, whether the dhMCM was functional, we expressed just Cdc6-E224Q (or Cdc6-WT) in a 2-hr window from M- to G1-phase and then monitored S-phase progression after switching off Cdc6 (mutant or WT) expression to allow Cdc6 removal. Cdc6 is an unstable protein (t1/2 ≤ 5 min) (Piatti et al., 1995) and so we expected that glucose addition (which strongly represses any further Cdc6 expression) would be followed by the disappearance of Cdc6 protein. Cells were synchronized similarly as in Figure 3, except after release from the G2/M block in galactose at 37°C, glucose was added 90 min later to repress the Gal1 promoter (Figure 4A). When no additional *CDC6* allele was expressed after the mitotic release, cells arrested with a 1C DNA content for the duration of the experiment and at later time points some cells had a sub-G1 DNA content (Figure 4A, leftmost time course), indicative of the reductional mitosis seen in the absence of *CDC6* (Piatti et al., 1995). When wild-type Cdc6 was expressed, cells progressed into G1 and S-phase quickly between the 90- and 120-min time points. When wild-type Cdc6 expression was repressed by glucose addition at t = 120, Cdc6 protein gradually disappeared (Figure 4B) and cells cycled approximately once more during the experiment. In contrast, if Cdc6-E224Q was continually expressed, cells arrested in G1-phase after release from the G2/M block and remained arrested (Figure 4A, asterisks). As a control, continual expression of wild-type Cdc6 or the Cdc6-NQ protein from the Gal1 promoter did not cause a G1 arrest (Figure 4—figure supplement 1) as published previously for wild-type Cdc6 (Perkins and Diffley, 1998; Schepers and Diffley, 2001). Remarkably, if Cdc6-E224Q expression was instead repressed 90 min after release (t = 120), the protein was rapidly eliminated (Figure 4B, bottom), and these cells entered a single round of DNA replication as indicated by the substantial S-phase population 30 min after glucose addition (Figure 4A, rightmost time course). This could only occur if a large number of replication origins initiated DNA replication following Cdc6-E224Q removal.

**Figure 4—figure supplement 1.**
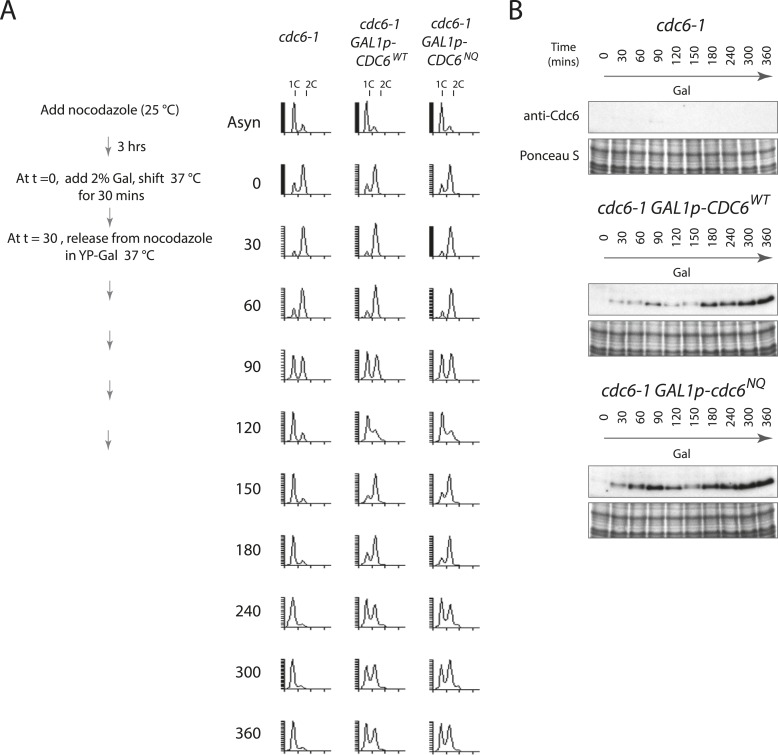
Overproduction of wild-type or Cdc6-NQ proteins does not cause a G1-arrest. (**A**) Cell synchronization protocol (right) and flow cytometry profiles (left) of yeast strains M378 (*cdc6-1*), M4455 (*cdc6-1 GAL1p-CDC6::LEU2*), and M4513 (*cdc6-1 GAL1p-cdc6-NQ::LEU2*). Asynchronous cells were arrested in G2/M for 3 hr with nocodazole, shifted up to 37°C for 30 min (from t = 0 to 30 min), and then released into G1-phase at 37°C expressing no additional Cdc6, *GAL1p-CDC6,* or *GAL1p-cdc6-NQ*. Cells overexpressing wild-type Cdc6 or Cdc6-NQ proteins proceed normally into S-phase. (**B**) Cdc6 Western blots (top panels) and total protein (bottom panels) by Ponceau S staining of the samples is shown in panel **A**. **DOI:** http://dx.doi.org/10.7554/eLife.05795.009

**Figure 5.**
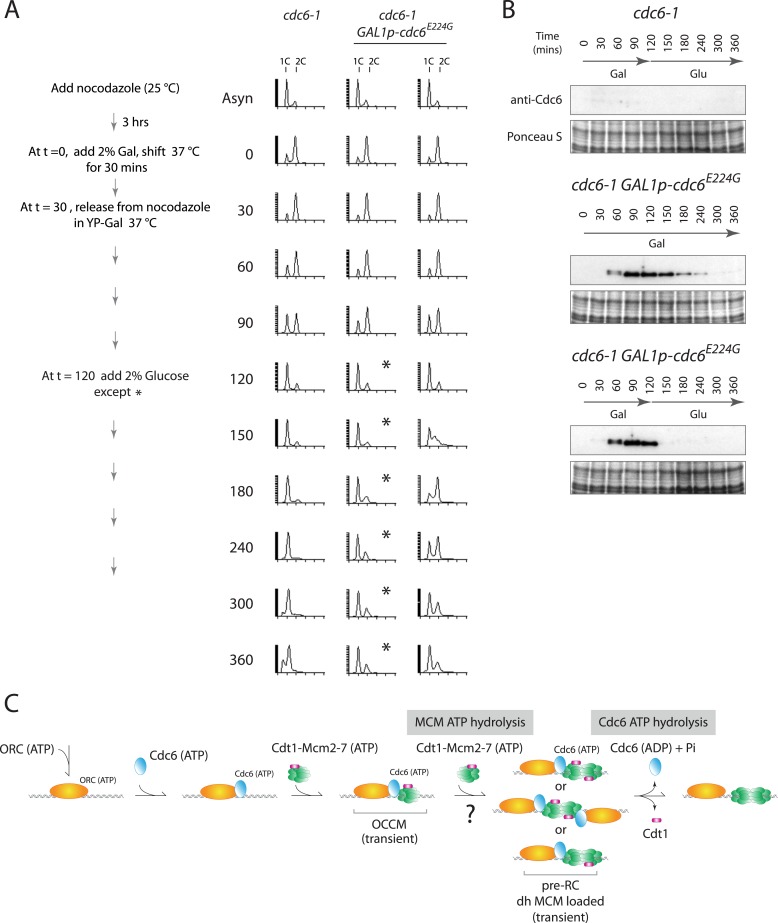
Cdc6-E224G ATPase mutant is also defective in G1 progression but its degradation promotes DNA replication. (**A**) Synchronization protocol (left) and flow cytometry profiles (right) of yeast strains M378 (*cdc6-1*) and M4766 (*cdc6-1 GAL1p-cdc6-E224G::LEU2*). Asynchronous cells were arrested in G2/M for 3 hr with nocodazole, shifted up to 37°C for 30 min (from t = 0 to 30 min), and then released into G1-phase at 37°C expressing no additional Cdc6 or *GAL1p-cdc6-E224G* as in Figure 4. (**B**) Cdc6 Western blots (top panels) and total protein (bottom panels) by Ponceau S staining of the samples shown in panel **A**. (**C**) Model for the role of Cdc6 ATP hydrolysis in DNA replication initiation (see text). **DOI:** http://dx.doi.org/10.7554/eLife.05795.010

This phenotype was not unique to the Cdc6-E224Q mutant. Recently, Cdc6-E224G has also been shown to efficiently load MCM in vitro using only purified proteins (Coster et al., 2014; Kang et al., 2014) and this allele is also defective in ATP hydrolysis (Randell et al., 2006; Speck and Stillman, 2007). We integrated this mutant into yeast under the control of the Gal1 promoter and repeated the experiment shown in Figure 4. When cells were released from the G2/M block continually expressing Cdc6-E224G (profile marked with asterisks in Figure 5A), they entered G1 phase and remained blocked in G1-phase like the *cdc6-1* control, indicating a failure to initiate DNA replication. Thus, *cdc6-E224G* behaves as a null mutant in agreement with the genetic analysis in Figure 1 and previous data (Perkins and Diffley, 1998). If *Gal1p-cdc6-E224G* was instead repressed 90 min after release from the nocodazole block (t = 120) and the protein was then degraded (Figure 5B bottom), cells entered S-phase and completely replicated DNA (Figure 5A, rightmost time course). Thus, the Cdc6-E224G mutant must also load MCM proteins in vivo (as it does in vitro), but subsequent steps in DNA replication initiation are blocked by its persistent expression.

**Figure 5—figure supplement 1.**
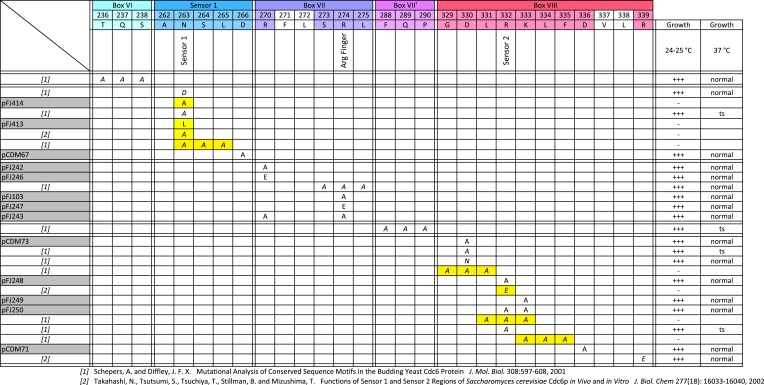
Growth phenotypes of *cdc6* mutants altering additional residues that potentially affect ATP hydrolysis or ATP sensing. Mutational summary of Box VI, sensor 1, Box VII (R-finger), Box VII', and Box VIII (sensor 2) regions of Cdc6. Yellow highlighting indicates that the mutation gave a lethal phenotype in yeast at 25°C. Plasmids from this study or previous published references for each mutant are listed at the left, and growth properties are listed at the right. **DOI:** http://dx.doi.org/10.7554/eLife.05795.011

**Figure 5—figure supplement 2.**
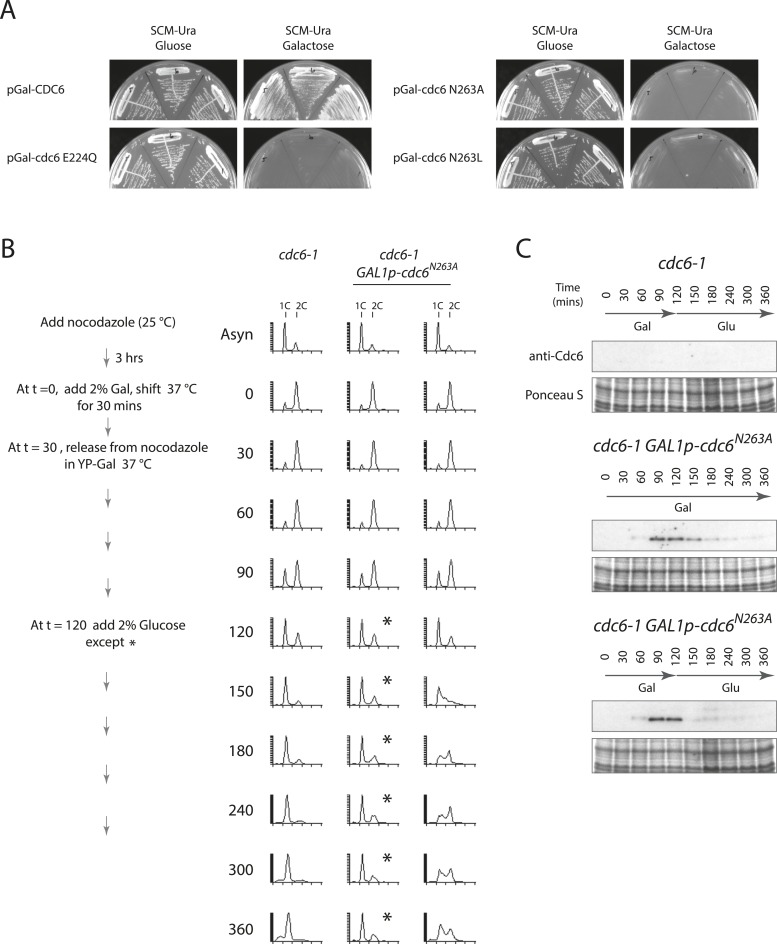
Expression of the Cdc6-N263A ATPase mutant protein is dominant negative for growth, causes a G1 block, but loads functional MCM that can promote DNA replication after Cdc6-N263A degradation. (**A**) pFJ235 (pRS416 *Gal1p-CDC6*) and mutant derivatives pFJ237 (*Gal1p-cdc6-E224Q*), pFJ404 (*Gal1p-cdc6-N263A*), and pFJ412 (*Gal1p-cdc6-N263L*) (Supplementary file 2) were transformed into wild-type yeast, W303-1A. Multiple transformants were streaked onto SCM-Ura plates containing glucose or galactose. (**B**) Cell synchronization protocol (left) and flow cytometry profiles (right) of yeast strains M378 (*cdc6-1*) and M4763 (*cdc6-1 GAL1p-cdc6-N263A::LEU2*). Asynchronous cells were arrested in G2/M for 3 hr with nocodazole, shifted up to 37°C for 30 min (from t = 0 to 30 min), and then released into G1-phase at 37°C expressing no additional Cdc6 or *GAL1p-cdc6-N263A*. *GAL1* promoter-driven *cdc6-N263A* expression was shut off at t = 120 min by the addition of glucose except where marked by an asterisk. (**C**) Cdc6 Western blots (top panels) and total protein (bottom panels) by Ponceau S staining of the samples are shown in panel (**B**). **DOI:** http://dx.doi.org/10.7554/eLife.05795.012

We constructed additional mutations in Cdc6 residues implicated in ATP hydrolysis or sensing bound nucleotide to further explore this function (Figure 5—figure supplement 1), which also lists previously published data for comparison. Single alanine substitutions of Cdc6 R270 or R274 (the conserved R-finger residue) gave no noticeable growth phenotype. Charge reversal mutations at these residues (R270E or R274E) also gave no phenotype indicating that these residues are not functioning as R-fingers. Mutation of charged residues to alanine in the conserved box VIII of AAA+ proteins including the R332 sensor 2 residue also gave no noticeable growth phenotype. In contrast, mutation of the sensor 1 asparagine residue (N263) to alanine or leucine gave a lethal phenotype and strong overproduction of the Cdc6 N263A or N263L mutant proteins from the Gal1 promoter gave a complete dominant negative growth phenotype (Figure 5—figure supplement 2; [Schepers and Diffley, 2001]), similar to the E224G or E224Q alleles. In summary, alanine or leucine mutations at the N263 sensor 1 residue were unique among the sensor 1, sensor 2, and ‘R-finger’ Cdc6 residues we tested in giving a lethal phenotype and both N263 mutant proteins were dominant negative for growth when over-expressed in wild-type yeast.

Since the N263A mutant is also defective for ATP hydrolysis (Speck and Stillman, 2007) but is capable of loading MCM proteins in vitro (Coster et al., 2014; Kang et al., 2014), we integrated this allele under the control of the Gal1 promoter in yeast and tested its role in DNA replication. Similar to the *cdc6-E224Q* and *cdc6-E224G* alleles, expression of only *cdc6-N263A* gave a growth arrest in G1 phase, but when the gene was repressed and the Cdc6-N263A protein subsequently degraded, cells entered S-phase (Figure 5—figure supplement 2).

Cdc6 ATPase activity is required for viability in budding yeast as seen by the phenotypes of multiple E224 and N263 substitution mutants (Figure 1; Figure 5—figure supplements 1, 2; and [Perkins and Diffley, 1998; Takahashi et al., 2002]). Using purified proteins, Cdc6 ATPase activity is not essential for MCM loading in vitro (Figure 2; and [Coster et al., 2014; Kang et al., 2014]) or in vivo (Figure 3) but is required for initiating DNA replication (Figures 4, 5). Since degradation of the ATPase defective Cdc6-E224Q, Cdc6-E224G and Cdc6-N263A mutant proteins after MCM loading promotes DNA replication (Figures 4, 5, and Figure 5—figure supplement 2), Cdc6 ATPase activity is required for Cdc6 release from the pre-RC, that is, an ORC-Cdc6 intermediate complex engaged with MCM (see model in Figure 5C). So, Cdc6 activity is not required after MCM loading other than for its removal from the helicase-loading complex. Since we did not trap an intermediate complex with ORC-Cdc6-Cdt1 and MCM double hexamer in vitro, we cannot conclude whether MCM hexamers are loaded in tandem, from two separate OCCM structures, or as a double hexamer. Since Cdc6-E224Q is defective for ATP hydrolysis and suppresses ORC ATP hydrolysis in the ORC-Cdc6-DNA context (Figure 2B), it appears that MCM ATP hydrolysis (Figure 2E) can drive the reaction forward in vitro to give double hexamer formation, albeit less efficiently that with wild-type Cdc6 (Figure 2C,D). However, since blocking Cdc6 ATP hydrolysis in vivo prevents subsequent MCM activation, this suggests that the in vitro reactions do not capture some essential aspect of this reaction. For instance, the dhMCM engaged with ORC-Cdc6-E224Q may be much more stable on origins within chromatin in yeast, which have a small 125-bp nucleosome-free region on average (Eaton et al., 2010).

We isolated multiple intragenic suppressors of the Cdc6-E224A or E224Q single mutants in the highly conserved ‘DEMD’ core region of the Walker B motif, that is, AAMD, AAAD, AAMA, NQMD, NQMA, and NQNM (Figure 1). All of these intragenic suppressors also mutate the first aspartic acid residue indicating that the presence of this D223 residue blocks Cdc6 activity in vivo when Cdc6 cannot hydrolyze ATP. The NQMD mutation did not restore Cdc6 ATPase activity (Figure 2B). This simplest explanation for the bypass phenotype is that a conformational change within the Cdc6-NQ mutant compensated for the loss of ATPase activity. This is supported by the fact that the AAMD, AAMA, AAAD mutants grew poorly but the NQMD and NQMN mutants, which have quite different substitutions at the ‘DE’ residues, grew very robustly at all temperatures (Figure 1—figure supplement 1). It is well established that ATP hydrolysis by AAA+ family members leads to conformational changes within these proteins, which drive the reactions forward, and this conformational change is communicated by residues in close proximity to the ATP/ADP binding pocket (Hanson and Whiteheart, 2005; Wendler et al., 2012; Duderstadt and Berger, 2013).

Recent work has discovered that the Cdc6 ATPase performs a quality control function in vitro (Coster et al., 2014; Kang et al., 2014). We suggest this is not its primary role in vivo since blocking a quality control function for the Cdc6 ATPase (i.e., ejecting incompletely assembled MCM hexamers or MCM-Cdt1 heptamers that bind the ORC-Cdc6 loader before loading onto dsDNA) should not give rise to a lethal phenotype. The hexameric replicative helicase structure and loading mechanisms have been honed over a long evolutionary history to be highly efficient (Forsburg, 2004; Bae et al., 2009; Slaymaker and Chen, 2012) and probably very few errors occur in vivo. Our data strongly suggest that the essential role of the Cdc6 ATPase is to disengage Cdc6 from the pre-RC after MCM is loaded. Interestingly, this role of the Cdc6 ATPase in initiating DNA replication is similar to bacterial helicase loaders: the AAA+ *Escherichia coli* helicase loader DnaC does not require ATP hydrolysis to load the hexameric DnaB helicase at *oriC*—but DnaC ATP hydrolysis leads to DnaC disassembly and release from a DnaC–DnaB complex that actually inhibits DnaB helicase activity (Davey et al., 2002; Arias-Palomo et al., 2013). Our data therefore reveal a broad conservation of replicative helicase loading mechanisms across kingdoms, which can hopefully be further elucidated by crystallographic or other biophysical methods using wild type and mutant helicase loading complexes.

## Materials and methods

### Construction of yeast strains and plasmids and yeast growth

Yeast strains and plasmids used in this study are listed in Supplementary files 1, 2. All strains were derivatives of W303-1A (*MAT***a** *ade2-1 trp1-1 can1-100 leu2-3,-112 his3-11,-15 ura3*). *GAL1-CDC6* genes were integrated into the *LEU2* locus as previously described (Weinreich et al., 1999). Genomic DNA from *LEU2+* transformants was isolated, digested with *Sal*I, and then probed by Southern blotting to identify single or double integrants. The presence of individual mutations was confirmed by sequencing the integrated *GAL1p-CDC6* allele. Single or double mutant integrant strains were chosen for further analysis based on which one gave the most similar amount of galactose-induced Cdc6 protein compared to the single wild-type *CDC6::LEU2* integrant, M4455. Point mutations and deletions within *CDC6* were generated by site-directed mutagenesis using the QuikChange system (Agilent Technologies, Santa Clara, CA). The wild-type *GAL1,10* promoter was cloned on a ∼800-bp *Eco*R1-*Bam*HI fragment into pRS416, and the wild-type *CDC6* coding sequence was cloned 39 bp downstream of the *GAL1,10* promoter cassette to give pFJ224. A potential 49-bp multi-stem loop structure that overlapped the first few codons of Cdc6 was removed by deleting 36 bp between the BamHI site on pFJ224 and the Cdc6 ATG, giving rise to pFJ235 (Figure 1—figure supplement 1). A SalI–SacI fragment of pFJ224 containing *pGAL1-CDC6* wild type was cloned into pRS405 to give pFJ304. The mutants pFJ305 (E224Q), pFJ306 (NQ), pFJ307 (NQMN), pFJ418 (N263A), and pFJ419 (E224G) were derived from pFJ304 by QuikChange. pGEX-CDC6 was described previously (Speck et al., 2005). The E224Q and DE(223,224)NQ mutations were introduced into pGEX-CDC6 using QuikChange to yield pFJ259 and pFJ263, respectively. Yeast cells were cultured in YPD (1% yeast extract, 2% peptone, 2% D-glucose) or synthetic complete medium (SCM) as described (Rose et al., 1990).

### Flow cytometry

The flow cytometry profiles of yeast cells were performed as described (Haase and Reed, 2002) with some modifications. Approximately 1 × 10^7^ cells were harvested, washed, and cell pellets were resuspended in 400 μl of water and fixed by adding 950 μl of 100% ethanol. Samples were kept at −20°C until further processed. Fixed cells were pelleted, washed with cold water, resuspended in 0.5 ml of 50 mM Tris pH 8, 15 mM NaCl containing 2 μg/ml RNase A, and incubated at 37°C overnight. Proteinase K was added to the samples for 1-hr 50°C incubation. Treated samples were pelleted again, resuspended in 0.5 ml 50 mM Tris pH 7.5, and sonicated to break-up cell clumps, if any. The samples were stained with SYTOX Green (Thermo Fisher, Grand Island, NY) at a final concentration of 2 μM and analyzed using a MoFlo Astrios (Beckman Coulter, Miami, FL).

### Chromatin immunoprecipitation

The Mcm2 ChIP assays were performed essentially as described (Pappas et al., 2004) using strains M378, M4455, M4464, M4513, and M4531. Overnight log cultures were diluted into fresh YP-Raf medium to a uniform OD and arrested with nocodazole for 3.5 hr. Galactose was added to a final 2%, and cells were shifted up to 37°C to inactivate *cdc6-1*. Cells were collected and resuspended in 37°C YP-Gal medium containing 5 μg/ml alpha factor and then treated with formaldehyde at the indicated time points. Monoclonal antibodies against Mcm2 (Mcm2-49, a gift of Bruce Stillman, Cold Spring Harbor Laboratory) were used for the ChIP assay after shearing chromatin to an average of ∼500 bp. The DNA primers used to amplify *ARS305* (310 bp) and surrounding regions are ‘305-350F’ GTCCCTGTAATTGGAAGAGC, ‘305-350R’ ACCACATAATGTGAAGCCTT, ‘305-310F’ ATGAGGTCTCTAGCAAAAAG, ‘305-310R’ TACTGTCCGGTGTGATTTAT, ‘305-239F’ TGAGCCTTCTAATAATAAAGGGGA, and ‘305-239R’ GTAACGTACCATTTTTGATCTTGG.

### Galactose-induction of Cdc6

The yeast strains M378, M4455, M4513, M4530, M4531, M4763, and M4766 were grown overnight in YP-Raffinose at 25°C and then treated with 15 μg/ml of nocodazole for 3 hr. 2% galactose (final) was then added, and cells were incubated at 37°C for a further 30 min. Nocodazole-arrested cells were washed once with water then released from the G2/M arrest in YP-Gal at 37°C. 2% glucose was added to the cultures at the indicated times unless stated otherwise.

### Pre-RC assay

The pre-RC assay was performed as described with minor modifications (Evrin et al., 2009). Here, a one-step reaction was used. 40 nM ORC, 80 nM (wt or mutant) Cdc6, 40 nM Cdt1, 40 nM MCM2-7 in buffer A (50 mM HEPES-KOH pH 7.5, 100 mM KGlu, 10 mM MgAc, 50 μM ZnAc, 3 mM ATP, 5 mM DTT, 0.1% Triton X-100, and 5% glycerol) were added to 6 nM linear pUC19-ARS1 DNA coupled to magnetic beads for 15 min at 24°C. Beads were washed 2 times with buffer A containing 300 mM KGlu plus 1 mM EDTA, or 2× short washes with buffer A-1 (50 mM HEPES-KOH pH 7.5, 1 mM EDTA, 300 mM KAc, 10% glycerol, 0.1% Triton X-100, and 5 mM DTT), or 3× with buffer B (50 mM HEPES-KOH pH 7.5, 1 mM EDTA, 500 mM NaCl, 10% glycerol, 0.1% Triton X-100, and 5 mM DTT) before digestion with 1 U of DNase I in buffer A plus 5 mM CaCl_2_ for 2 min at 24°C. The samples were separated by SDS-PAGE and analyzed by silver staining.

### Expression and purification of proteins

ORC was expressed by using baculovirus-infected cells and purified as described (Klemm et al., 1997). Cdc6 (WT and mutants) and Cdt1 were expressed in bacteria and purified as described (Speck et al., 2005; Evrin et al., 2009). Mcm2-7 were expressed in *Saccharomyces cerevisiae* and purified as described (Evrin et al., 2009).

### In vitro assembly and purification of the OCCM and Mcm2-7 double-hexamer complexes for single particle EM

The pre-RC were assembled in a one-step reaction: 40 nM ORC, 80 nM Cdc6, 40 nM Cdt1, and 40 nM MCM2–7 in buffer A (50 mM HEPES–KOH pH 7.5, 100 mM KGlu, 10 mM MgAc, 50 mM ZnAc, 3 mM ATP, 5 mM DTT, 0.1% Triton X-100, and 5% glycerol) were added to 6 nM pUC19-ARS1 plasmid beads at 24°C (Evrin et al., 2009). After 10 min (for pre-RC intermediate analysis) or 40 min (for MCM2-7 double-hexamer analysis), the beads were washed 3 times with buffer A (pre-RC intermediate) or B (MCM2-7 double-hexamer) and 3 times with buffer C (50 mM HEPES-KOH [pH 7.5], 100 mM potassium acetate, 5 mM magnesium acetate, 5 mM CaCl_2_) and eluted with 1 U DNase I in 5 µl buffer C.

### ATPase assay

The ATPase assay was performed as described (Speck and Stillman, 2007; Fernandez-Cid et al., 2013). Error bars represent the standard deviation from at least three independent experiments.

### EM grids preparation

The concentration of pre-RC intermediates and loaded dhMCM was low for cryo-EM. To get more particles in each image, we added a thin continuous carbon on top of the commercially available lacey carbon grids (SPI #3830C-MB). We coated a very thin carbon layer on freshly cleaved mica using an Edwards Auto 306 evaporator, floated the thin carbon film off the mica surface onto water surface, then lowered the water level to deposit the carbon film on lacey grids. We glow-discharged the dried grids in a PELCO easiGlow before applying 3 µl sample onto the surface. After blotting for 5 s, we plunged the grid into liquefied ethane to get vitrified sample in the FEI Vitrobot, keeping the sample chamber temperature at 11°C, relative humidity 90%, and offset position of −1 mm. For negative stain EM grids preparation, we first glow-discharged the carbon-coated EM grids in PELCO easiGlow, applied 3 µl sample, blotted the grids with a piece of filter paper, applied one drop of 1% uranyl acetate solution, waited about 30 s, blotted and applied another drop of stain solution, waited about 1 min, then blotted to nearly but not completely dry, and left a thin layer of stain solution on the grid for air drying.

### Electron microscopy and single particle image analysis

JEM-2010F TEM with a Gatan 626 cryo-holder was used for both negative stain and cryo EM grids observation. Micrographs were recorded with an electron dose of 15 e^−^/Å^2^ at a magnification of 50,000 in a 4k × 4k Gatan Ultrascan CCD camera. EMAN2.1 package (Tang et al., 2007) was used for image processing. We first manually picked raw particle images with e2boxer, then did contrast transfer function correction following EMAN2 document. The phase flipped particles were pooled into one image stack, shrunk by a factor of 2, and computationally classified and averaged. Each image class had at least 10 particles for negative-stained samples and 20 for cryo samples. The defocus range was −0.5 to −4 μm. The image pixel size was 4.23 Å.

## Additional files

Supplementary file 1. Yeast strains used in this study. **DOI:** http://dx.doi.org/10.7554/eLife.05795.013

Supplementary file 2. Plasmids used in this study. **DOI:** http://dx.doi.org/10.7554/eLife.05795.014
