# Supplementary material for: Cdc6 ATPase activity disengages Cdc6 from the pre-replicative complex to promote DNA replication
Source: eLife. 2015 Aug 25;4:e05795. doi: 10.7554/eLife.05795 (PMC4547096; doi:10.7554/eLife.05795)
Supplement: Supplementary file 1. — Yeast strains used in this study. DOI: http://dx.doi.org/10.7554/eLife.05795.013 [file elife05795s001.docx]

Supplementary File 1 Yeast strains used in this study

| Strain | Genotype | Reference |
| --- | --- | --- |
| W303-1A | *MATa ade2-1 ura3-1 his3-11,15 trp1-1 leu2-3,112 can1-100* | R. Rothstein |
| K4055 | *ade2-1 ura3-1 leu2-3,112 his3-11,15 can1-100 cdc6∆::hisG-ura3-hisG trp1-1::pMET3-CDC6::TRP1* | K. Nasmyth |
| M23 | W303-1A *cdc6∆::ura3 x pRS426-CDC6* | M. Weinreich |
| M378 | W303-1A *cdc6-1* | M. Weinreich |
| M4455 | W303-1A *cdc6-1 GAL1p-CDC6::LEU2 (1x)* | This study |
| M4464 | W303-1A *cdc6-1 GAL1p-cdc6-NQMN::LEU2 (1x)* | This study |
| M4466 | W303-1A *cdc6∆::ura3 x pRS416-CDC6* | This study |
| M4513 | W303*-*1A *cdc6-1 GAL1p-cdc6-NQMD::LEU2 (1x)* | This study |
| M4530 | W303-1A cdc6-1 *GAL1p-cdc6-E224Q::LEU2 (1x)* | This study |
| M4531 | W303-1A *cdc6-1 GAL1p-cdc6-E224Q::LEU2 (2x)* | This study |
| M4758 | K4055 *x pMW71* | This study |
| M4759 | K4055 *x pRS415* | This study |
| M4760 | K4055 *x pFJ21* | This study |
| M4762 | K4055 *x pFJ230* | This study |
| M4763 | W303-1A *cdc6-1 GAL1p-cdc6-N263A::LEU2 (2x)* | This study |
| M4766 | W303-1A *cdc6-1 GAL1p-cdc6-E224G::LEU2 (2x)* | This study |
