# Supplementary material for: Cdc6 ATPase activity disengages Cdc6 from the pre-replicative complex to promote DNA replication
Source: eLife. 2015 Aug 25;4:e05795. doi: 10.7554/eLife.05795 (PMC4547096; doi:10.7554/eLife.05795)
Supplement: Supplementary file 2. — Plasmids used in this study. DOI: http://dx.doi.org/10.7554/eLife.05795.014 [file elife05795s002.docx]

Supplementary File 2 Plasmids used in this study

| Plasmid | Description |
| --- | --- |
| pRS415 | ARS CEN LEU2 vector |
| pRS416 | ARS CEN URA3 vector |
| pRS405 | LEU2 vector (for integration into chromosome) |
| pMW71 | pRS415-CDC6 |
| pMW369 | pRS415-cdc6 DE(223,224)AA |
| pMW388 | pRS415-cdc6 E224G (GGG) |
| pCDM67 | pRS415-cdc6 D266A |
| pCDM71 | pRS415-cdc6 D336A |
| pCDM73 | pRS415-cdc6 D330A |
| pGEX-CDC6 | GST-CDC6 fusion on pGEX-61 |
| pFJ15 | pRS415-cdc6 D223A |
| pFJ16 | pRS415-cdc6 E224A (GCG) |
| pFJ17 | pRS415-cdc6 M225A |
| pFJ18 | pRS415-cdc6 D226A |
| pFJ19 | pRS415-cdc6 DEMD(223,224,225,226)AAAA |
| pFJ20 | pRS415-cdc6 E224L |
| pFJ21 | pRS415-cdc6 E224Q |
| pFJ22 | pRS415-cdc6 MD(225,226)AA |
| pFJ24 | pRS415-cdc6 AEAA |
| pFJ72 | pRS415-cdc6 AAAD |
| pFJ73 | pRS415-cdc6 AAMA |
| pFJ101 | pRS415-cdc6 V221A |
| pFJ102 | pRS415-cdc6 L228A |
| pFJ103 | pRS415-cdc6 R274A |
| pFJ108 | pRS415-cdc6 L222A |
| pFJ115 | pRS415-cdc6 DAAA |
| pFJ216 | pRS416-GAL1p-NΔ49-CDC6 (=pFJ224 except deleting Cdc6 residues 2-49) |
| pFJ217 | pRS416-GAL1p-NΔ49-cdc6-E224G (=pFJ225 except deleting Cdc6 residues 2-49) |
| pFJ223 | pRS416-GAL1p-NΔ49-cdc6-E224Q (=pFJ226 except deleting Cdc6 residues 2-49) |
| pFJ224 | pRS416-GAL1p-CDC6 |
| pFJ225 | pRS416-GAL1p-cdc6-E224G |
| pFJ226 | pRS416-GAL1p-cdc6-E224Q |
| pFJ230 | pRS415-cdc6 NQMD |
| pFJ235 | pRS416-GAL1p-Δ36-CDC6 (deleted 36bp prior to codon 1 on pFJ224 to remove predicted stem loop) |
| pFJ236 | pRS416-GAL1p-Δ36-cdc6-E224G (as pFJ235 except E224G) |
| pFJ237 | pRS416-GAL1p-Δ36-cdc6-E224Q (as pFJ235 except E224Q) |
| pFJ242 | pRS415-cdc6 R270A |
| pFJ243 | pRS415-cdc6 R270A R274A |
| pFJ246 | pRS415-cdc6 R270E |
| pFJ247 | pRS415-cdc6 R274E |
| pFJ248 | pRS415-cdc6 R332A |
| pFJ249 | pRS415-cdc6 K333A |
| pFJ250 | pRS415-cdc6 RK(332,333)AA |
| pFJ257 | pRS415-cdc6 NQMA |
| pFJ258 | pRS415-cdc6 NQMN |
| pFJ259 | pGEX-cdc6 E224Q |
| pFJ263 | pGEX-cdc6 NQMD |
| pFJ304 | pRS405-GAL1p-CDC6 (integration plasmid) |
| pFJ305 | pRS405-GAL1p-cdc6 E224Q (integration plasmid) |
| pFJ306 | pRS405-GAL1p-cdc6 NQ (integration plasmid) |
| pFJ307 | pRS405-GAL1p-cdc6 NQMN (integration plasmid) |
| pFJ360 | pRS415-cdc6 E224G (GGA) |
| pFJ361 | pRS415-cdc6 E224A (GCA) |
| pFJ404 | pFJ235 GAL1p-cdc6-N236A |
| pFJ412 | pFJ235 GAL1p-cdc6-N236L |
| pFJ413 | pRS415-cdc6 N263L |
| pFJ414 | pRS415-cdc6 N263A |
| pFJ418 | pRS405-GAL1p-cdc6 N263A (integration plasmid) |
| pFJ419 | pRS405-GAL1p-cdc6 E224G (integration plasmid) |
